# Pessimistic outcome expectancy does not explain ambiguity aversion in decision-making under uncertainty

**DOI:** 10.1038/s41598-019-48707-y

**Published:** 2019-08-21

**Authors:** C. Ahrends, F. Bravo, M. L. Kringelbach, P. Vuust, M. A. Rohrmeier

**Affiliations:** 1 Center for Music in the Brain, Department of Clinical Medicine, Aarhus University & The Royal Academy of Music Aarhus/Aalborg, Aarhus, Denmark; 2 Cognition and Consciousness Imaging Group, Division of Anaesthesia, Wolfson College, University of Cambridge, Cambridge, UK; 3 Hedonia Research Group, Department of Psychiatry, University of Oxford, Oxford, UK; 4 Digital and Cognitive Musicology Lab, Digital Humanities Institute, École Polytechnique Fédérale de Lausanne, Lausanne, Switzerland

**Keywords:** Decision, Human behaviour

## Abstract

When faced with a decision, most people like to know the odds and prefer to avoid ambiguity. It has been suggested that this aversion to ambiguity is linked to people’s assumption of worst possible outcomes. We used two closely linked behavioural tasks in 78 healthy participants to investigate whether such pessimistic prior beliefs can explain ambiguity aversion. In the risk-taking task, participants had to decide whether or not they place a bet, while in the beliefs task, participants were asked what they believed would be the outcome. Unexpectedly, we found that in the beliefs task, participants were not overly pessimistic about the outcome in the ambiguity condition and in fact closer to optimal levels of decision-making than in the risk conditions. While individual differences in pessimism could explain outcome expectancy, they had no effect on ambiguity aversion. Consequently, ambiguity aversion is more likely caused by general caution than by expectation of negative outcomes despite pessimism-dependent subjective weighting of probabilities.

## Introduction

Probabilistic decision-making is a crucial ability to navigate every-day situations that involve uncertainty about an outcome. Decades of research from different disciplines have tried to explain the way in which these decisions are formed. As opposed to traditionally presumed linear models of decision-making^1,2^, economic and general psychological theories have suggested that humans often do not show optimal performance to maximise their reward value^3–7^. Especially the question of how decisions are made under uncertainty has been controversially debated. For example, Ellsberg^8^ has proposed that the level of information individuals have in a given situation influences the way they make a decision. He defines ambiguity and risk as two forms of uncertainty: ambiguity as not knowing the odds and risk as knowing the odds. He describes a situation where participants have to place a bet on either one of two urns: one containing 50 red balls and 50 black balls and one containing 100 red and black balls of which the distribution is unknown. Ellsberg shows how participants in the experiment prefer to place bets on both the red balls and the black balls from the first urn to either option from the second urn. This implies that they consider both options from the second urn to be less likely than both options from the first urn (which they know to be 0.5) – a paradox, given that the probabilities always sum to 1. With this paradox, Ellsberg introduces the idea of ambiguity aversion resulting in potentially suboptimal choices. In the subsequent decades, this question has been empirically tested and controversially discussed. The evolution of these theories and their evidence is reviewed e.g., in Etner, *et al*.^9^. For a comparison of normative, descriptive, and computational approaches to decision-making under uncertainty, see Johnson and Busemeyer^10^.

The dissociation of ambiguity and risk is particularly interesting in the context of predictive coding. Predictive coding is a highly prominent framework that unifies perceptive and cognitive brain processes^11–16^. The idea of predictive coding is related to the broad group of Bayesian theories of decision-making. These approaches model the decision-making process as updating prior beliefs by sampling from available information^17,18^. According to this theory, higher levels of the brain are constantly making predictions about the environment relying on prior information that are passed down to lower (perceptual) levels. The sensory signal is then compared with the brain’s predictions and the resulting error passed back to update information on the higher levels. One main question in this context is how a prior is formed if the initial information is rudimentary or ambiguous, making it impossible to make a good prediction^19–21^. A potential strategy is to take information other than the one strictly related to the decision into account. It has been shown that, besides objective probabilities, decision-making is influenced by subjective factors related to the individual and the situation, like personality, affective state, or framing^4,22–24^. At first glance, this subjective weighting of probabilities might seem inaccurate, but it can be a useful strategy to improve the individual’s chances of reward or reduce negative emotions. For instance, a pessimistic attitude towards a personal outcome can protect from disappointment by fulfilling the expectation in the case of failure – a phenomenon known as “bracing”^25^. However, both optimism and pessimism can be adaptive strategies for decision-making: While optimism increases reward-seeking behaviour, pessimistic bracing prepares the individual for an undesired outcome^25^. It has been suggested that the balance of optimism and pessimism is strongly linked to the expectation of consequences in that only close, personally relevant feedback leads to bracing^26^.

**Figure 1:**
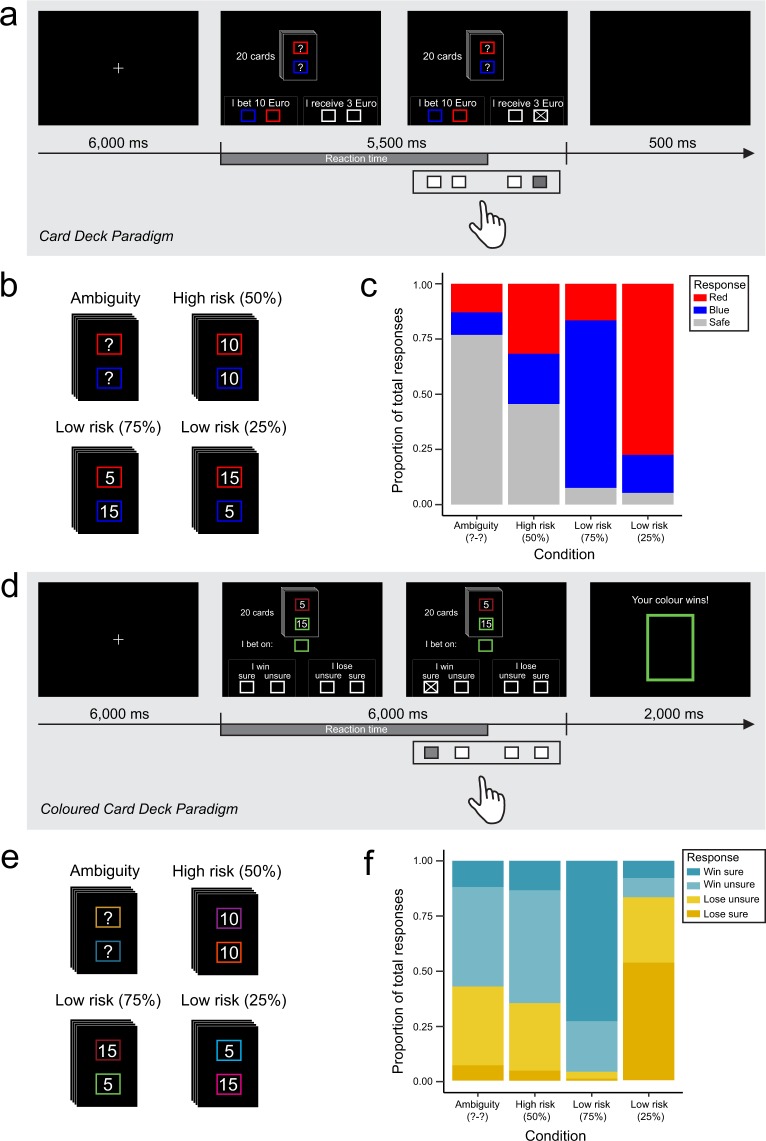
Experimental design for Version A, Card Deck Paradigm, (**a**–**c**) and Version B, Coloured Card Deck Paradigm, (**d**–**f**). (**a**) Shows an example of an experimental trial of the Card Deck Paradigm. After an initial fixation cross (only between blocks), participants see an image of a deck of cards. In the example, there is an unknown amount of red cards and an unknown amount of blue cards in the deck (ambiguity condition). At the bottom of the screen, participants can choose to place a bet (left) on either blue or red or opt out (right). Participants have 5500 milliseconds (time-out) to make a decision. Reaction time is measured within this period from the onset of the stimulus until the moment of the button-press. (**b**) Illustrates the experimental conditions ambiguity, high risk, and low risk (divided into 75% and 25%). (**c**) Shows the responses as proportions of the total amount grouped by condition. (**d**) Gives an example of an experimental trial of the Coloured Card Deck Paradigm. The trial specifies the low risk (75%) condition with 5 dark red cards and 15 green cards. Participants are assigned a colour, in this case green, which gives them a 75% chance of winning. At the bottom of the screen, they indicate if they believe they are going to win (left) or lose (right), as well as how sure they are about their guess (“sure” or “unsure” in each field). Participants have 6000 milliseconds to make a decision. After the time-out, the outcome of each trial is revealed for 2000 milliseconds. (**e**) Represents the experimental conditions. The conditions are defined in each trial by the amount of cards from each colour in combination with the colour assignment underneath the card deck. The position (upper or lower panel) of the assigned colour was randomised within each pair. (**f**) Shows the responses as proportions grouped by condition.

To explain ambiguity aversion as described by the Ellsberg paradox, researchers have suggested that people are generally pessimistic and therefore assume their chances of winning inferior to their chances of losing when they do not know the probabilities^27–29^. Other studies suggest, however, that people are generally optimistic about personal outcomes^30^ or that, instead of global optimism or pessimism, individual differences in attitude explain the weighting of probabilities under ambiguity^24^. The simple logic underlying these arguments is that optimism and pessimism respectively equal positive and negative outcome expectancy and that the reason for ambiguity aversion is that people expect more negative outcomes^28^. The difference between decision-making under risk and under ambiguity can then be interpreted as a stronger influence of pessimistic outcome expectancy on ambiguous choices to compensate for the missing information about probabilities. Here, we approached this theory experimentally by comparing two similar paradigms in the same sample of participants but with different questions. To this aim, we establish the *Coloured Card Deck Paradigm*, a new version of the *Card Deck Paradigm*^31^, to investigate outcome expectancy in decision-making under uncertainty. The first part of the study, an adaptation of the *Card Deck Paradigm*, assesses ambiguity aversion in a bet/opt-out task. In the second part, we employed the *Coloured Card Deck Paradigm*, which uses the same settings, but asks participants to guess whether, given the specified or unspecified probabilities, they believe they are going to win or lose. To provide further information about prior beliefs, we also asked whether they feel sure or unsure about this decision. Both paradigms are illustrated in Fig. 1. For details on the two paradigms and the data acquisition, please see the Methods section. In line with the afore-mentioned theories, we hypothesised that participants choose the opt-out option more often under ambiguity than under risk in the first version of the paradigm. Furthermore, if pessimism is the underlying cause of ambiguity aversion, we expect participants to have more pessimistic expectations about their outcome (i.e., consider their chances of winning inferior to 50%) in the ambiguity condition in the second version of the paradigm. Besides general pessimism, we hypothesised that individual differences in pessimism have both explanatory and predictive value for outcome expectations and ambiguity aversion. In line with the Dual Systems Theory^3^, we hypothesise that reaction times in the paradigm reflect the level of uncertainty with longest reaction times for the ambiguity condition and shortest reaction times for the low risk condition.

## Results

### Logistic regression modelling

Model specification:

Y = Response (binary option bet/safe); x_1 _= Uncertainty; ε = Random effects (Participant)

$${{\rm{A}}}_{-}{{\rm{GLM}}}_{{\rm{0}}}:\,P(Y)=\frac{1}{1+{e}^{-({\theta }_{0}+\varepsilon )}\,}$$

$${{\rm{A}}}_{-}{{\rm{GLM}}}_{{\rm{1}}}:\,P(Y)=\frac{1}{1+{e}^{-({\theta }_{0}+{\theta }_{1}{x}_{1}+\varepsilon )}\,}$$

**Figure 2:**
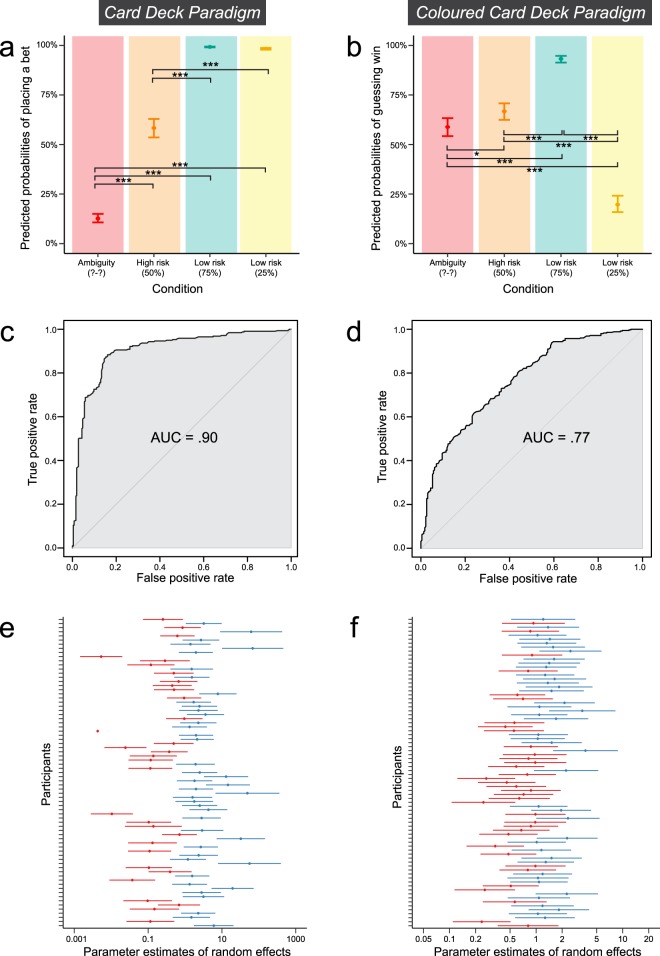
Model output and diagnostics for A_GLM_1_ and B_GLM_1_. (**a**,**b**) Predicted probabilities (y-axis) of the models grouped by condition (x-axis). The error bars show the 95% confidence interval of predicted probabilities for the fixed effects computed from the whole models (taking random effects into account). The significance levels indicated by the asterisks represent Bonferroni-corrected least square means comparisons. (**a**) Shows the predicted probabilities of placing a bet in Version A of the paradigm obtained from model A_GLM_1_. (**b**) Shows the predicted probabilities of guessing “win” in Version B of the paradigm as obtained from model B_GLM_1_. (**c**,**d**) Show the ROC-plots of the models as a measure of model performance. The curves plot the true positive rates of the model on the y-axis against the false positive rates on the x-axis as computed on the test set for different discrimination thresholds. A curve leaning towards the top left, away from the diagonal, indicates a good model performance and a curve leaning towards the bottom right would point towards a poorer model performance with more false positives and false negatives. The integral of the curve (AUC) is an additional measure of model performance. (**c**) Shows the ROC-plot for model A_GLM_1_ as obtained by testing the model on the test set for Version A of the paradigm. (**d**) Represents the ROC-curve for model B_GLM_1_ as tested on the test set for Version B of the paradigm. (**e**,**f**) Plot the estimates of random effects of the mixed models together with their 95% confidence intervals with all participants on the y-axis and the estimates on the x-axis. (**e**) Shows the random effects in model A_GLM_1_. (**f**) Plots the random effects in model B_GLM_1_.

Both models were fit to the training set of the data consisting of 1669 observations (*n* = 77). The binomial null model A_GLM_0_ was significantly improved by the factor uncertainty (A_GLM_1_). This indicates that the level of uncertainty has a significant effect on responses. The detailed results of the model comparisons can be found in Table 1. Both the Akaike Information Criterion (AIC) and the Bayes Information Criterion (BIC) decrease from model A_GLM_0_ to A_GLM_1,_ indicating that the improvement in model fit outweighs added complexity. Supplementary Table S2 shows obtained weights and odds ratios together with their confidence intervals from the resulting model A_GLM_1_. The predicted probabilities to place a bet based on the model are illustrated in Fig. 2a. The figure shows that the differences between risk and ambiguity in probabilities of placing a bet are larger than in the previous study (original values: 48.27% certain payoff choices in the ambiguity condition and 39.83% in the risk condition)^31^. The ambiguity condition produces lowest mean probabilities of placing a bet (21.55%), the high risk condition 55.42% and the low risk condition (subdivisions 75% and 25%) at the highest end towards 100% mean probability of placing a bet (95.08% and 96.36%, respectively). The confidence intervals in the low risk condition are very small indicating little variability in this condition. On the test set, the resulting model reaches 86.01% accuracy (above chance 50%), indicating that the model evaluation was successful. The true and false positive rates of the performance on the test set with different discrimination thresholds are shown in the receiver operating characteristics (ROC)-plot in Fig. 2c with an area under the curve (AUC) of 0.90. The figure indicates a very good model performance with a strong tendency towards the top left corner. The illustrated integral (AUC) further supports good model performance. The random effects included in the model have a standard deviation of 2.15 and an intraclass correlation coefficient (ICC) of 0.58. They are plotted in Fig. 2e. The wideness of the plot indicates that there was high individual variability in the data. The plot also identifies one participant (single red dot) with a confidence interval of 0 meaning that this participant gave the same response in all trials. For details on the analysis steps including model fitting, please see the methods section.

**Table 1:** Binomial logistic regression model comparisons for Version A and Version B.

Model	Log Likelihood	AIC	BIC	Pseudo-R^2^	Likelihood ratio test
A_GLM_0_	−1048.99	2102.0	2112.8	0.23	Model χ^2^(3) = 853.04, *P* < .001
A_GLM_1_	−622.47	1254.9	1282.0	0.78	
B_GLM_0_	−1151.5	2307.0	2318.0	0.08	Model χ^2^(3) = 363.49, *P* < .001
B_GLM_1_	−969.8	1949.5	1976.8	0.36	
B_GLMc_0_	−1078.7	2161.3	2172.3	0.15	Model χ^2^(3) = 465.76, *P* < .001
B_GLMc_1_	−845.8	1701.7	1728.9	0.47

The same models were applied to Version B of the paradigm:

Y = Guess (binary option win/lose); x_1_ = Uncertainty; ε = Random effects (Participant)

$${{\rm{B}}}_{-}{{\rm{GLM}}}_{{\rm{0}}}:\,P(Y)=\frac{1}{1+{e}^{-({\theta }_{0}+\varepsilon )}\,}$$

$${{\rm{B}}}_{-}{{\rm{GLM}}}_{{\rm{1}}}:P(Y)=\frac{1}{1+{e}^{-({\theta }_{0}+{\theta }_{1}{x}_{1}+\varepsilon )}\,}$$

The models were fit to the training set of the data consisting of 1742 observations (*n* = 77). Also in the new version of the paradigm, the null model B_GLM_0_ predicting guesses was significantly improved by the factor uncertainty B_GLM_1_ (see Table 1). Both AIC and BIC decrease for model B_GLM_1_, indicating good model fit. Supplementary Table S3 contains the obtained odds ratios and weights from model B_GLM_1_. Figure 2b shows the probabilities of guessing “win” in each uncertainty condition, as predicted by the model. The optimal decisions for each condition would be at 50% for both the ambiguity and the high risk condition, at 75% for low risk (75%) condition and at 25% for the low risk (25%) condition. While this general picture can be found in the plotted probabilities, the ambiguity condition is closer to the optimal level of 50% (with a mean probability of 58.84%) than the high risk condition, which produces overly optimistic responses at 65.22%. Also the low risk (75%) condition is overly optimistic with 92.74% mean probability of guessing win, whereas the low risk (25%) condition produces slightly pessimistic responses (mean 21.28%). Notably, the predicted probability for the ambiguity condition is not below 50%, which does not support the notion of pessimistic priors. When tested on the test set, the model predicted with an accuracy of 71.13% (above chance 50%). The true and false positive rates are illustrated in Fig. 2d with an AUC of .77. While the model performance is less optimal than the one for Version A, it is leaning away from the diagonal towards the top left corner, indicating an acceptable performance and a large integral (AUC). The random effects of this model have a standard deviation of 0.91 and an ICC of 0.20 (see Fig. 2f). The plot shows that the individual variability is overall smaller than in model A_GLM_1_.

We then tested whether the factor uncertainty can explain and predict confidence ratings in Version B of the paradigm. For this part, the models were specified as follows:

Y = Confidence (binary option sure/unsure); x_1_ = Uncertainty; ε = Random effects (Participant)

$${{\rm{B}}}_{-}{{\rm{GLMc}}}_{{\rm{0}}}:\,P(Y)=\frac{1}{1+{e}^{-({\theta }_{0}+\varepsilon )}\,}$$

$${{\rm{B}}}_{-}{{\rm{GLMc}}}_{{\rm{1}}}:\,P(Y)=\frac{1}{1+{e}^{-({\theta }_{0}+{\theta }_{1}{x}_{1}+\varepsilon )}\,}$$

Like the model predicting guesses, also the confidence null model (B_GLMc_0_) was significantly improved by the factor uncertainty (B_GLMc_1_). Model comparisons are summarised in Table 1. The details of the obtained parameters can be found in Supplementary Table S4.

### Post-hoc comparisons

In order to confirm which conditions are significantly different from each other, we performed pairwise comparisons using least square means with the probabilities obtained from the model. The results can be found in Table 2. Supporting the main hypothesis, the differences between placed bets in the ambiguity condition and high risk (50%) condition were highly significant. Responses in both of these conditions were significantly different from the low risk condition.

**Table 2:** Least square mean comparisons for models A_GLM_1_, B_GLM_1_, and B_GLMc_1_.

Model	Contrast	95% Confidence Interval for Odds Ratio			Standard Error	Z-ratio	P-value
		*Lower*	*Odds Ratio*	*Upper*			
A_GLM_1_	High risk (50%) – Ambiguity (?-?)	5.94	9.64	15.64	1.77	12.36	<0.0001
	High risk (50%) – Low risk (25%)	0.00	0.01	0.03	0.00	−10.14	<0.0001
	High risk (50%) – Low risk (75%)	0.01	0.02	0.06	0.01	−10.12	<0.0001
	Ambiguity (?-?) – Low risk (25%)	0.00	0.00	0.00	0.00	−14.22	<0.0001
	Ambiguity (?-?) – Low risk (75%)	0.00	0.00	0.01	0.00	−14.88	<0.0001
	Low risk (25%) – Low risk (75%)	0.67	2.24	7.50	1.03	1.76	0.47
B_GLM_1_	High risk (50%) – Ambiguity (?-?)	1.00	1.40	1.97	0.18	2.64	0.05
	High risk (50%) – Low risk (25%)	5.14	8.19	13.06	1.45	11.90	<0.0001
	High risk (50%) – Low risk (75%)	0.08	0.15	0.27	0.03	−8.05	<0.0001
	Ambiguity (?-?) – Low risk (25%)	3.69	5.84	9.24	1.02	10.12	<0.0001
	Ambiguity (?-?) – Low risk (75%)	0.06	0.10	0.19	0.02	−9.50	<0.0001
	Low risk (25%) – Low risk (75%)	0.01	0.02	0.04	0.00	−14.87	<0.0001
B_GLMc_1_	High risk (50%) – Ambiguity (?-?)	0.86	1.34	2.07	0.22	1.75	0.48
	High risk (50%) – Low risk (25%)	0.06	0.10	0.16	0.02	−12.75	<0.0001
	High risk (50%) – Low risk (75%)	0.04	0.06	0.11	0.01	−14.17	<0.0001
	Ambiguity (?-?) – Low risk (25%)	0.04	0.07	0.12	0.01	−13.82	<0.0001
	Ambiguity (?-?) – Low risk (75%)	0.03	0.05	0.08	0.01	−15.15	<0.0001
	Low risk (25%) – Low risk (75%)	0.40	0.67	1.12	0.13	−2.05	0.24

P-values and confidence levels are adjusted using Bonferroni correction. Odds ratios represent increases (values > 1) and decreases (values < 1) in odds of placing a bet for model A_GLM_1_, odds of guessing “win” for model B_GLM_1_, and odds of responding “sure” for model B_GLMc_1_, respectively.

The same test was applied to the model predicting guesses (B_GLM_1_) and the model predicting confidence (B_GLMc_1_) obtained for the new version of the paradigm. The results are reported in Table 2. We found a significant difference in “win” guesses between high risk (50%) and ambiguity. As expected, also the difference between 75% risk and 25% risk were significant. Notably, the contrast of confidence ratings between high risk (50%) and ambiguity was not significant.

### Pessimism model fitting

Model specification for A_GLM, B_GLM, and B_GLMc:

Y = Response (Bet, Guess, and Confidence, respectively); x_1_ = Uncertainty; x_2_ = Pessimism; ε = Random effects (Participant)

$${{\rm{Model}}}_{{\rm{0}}}:\,P(Y)=\frac{1}{1+{e}^{-({\theta }_{0}+\varepsilon )}\,}$$

$${{\rm{Model}}}_{{\rm{1}}}:\,P(Y)=\frac{1}{1+{{\rm{e}}}^{-({{\theta }}_{0}+{{\theta }}_{1}{{\rm{x}}}_{1}+{\varepsilon })}\,}$$

$${{\rm{M}}{\rm{o}}{\rm{d}}{\rm{e}}{\rm{l}}}_{2}:\,P(Y)=\frac{1}{1+{{\rm{e}}}^{-({\theta }_{0}+{\theta }_{1}{{\rm{x}}}_{1}+{\theta }_{2}{{\rm{x}}}_{2}+\varepsilon )}\,}$$

**Figure 3:**
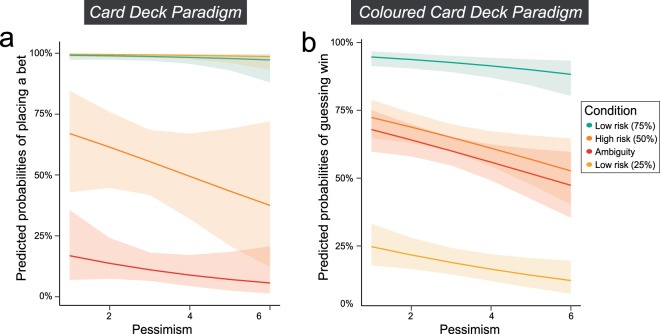
Fixed effect pessimism in models A_GLM_2_ and B_GLM_2_. (**a**,**b**) Predicted probabilities (y-axis) of the models by pessimism scores (x-axis) and grouped by condition (colours). The shading represents the respective 95% confidence interval of predicted probabilities for the fixed effects computed from the whole models (taking random effects into account). (**a**) Shows the predicted probabilities of placing a bet in Version A of the paradigm obtained from model A_GLM_2_. (**b**) Shows the predicted probabilities of guessing “win” in Version B of the paradigm as obtained from model B_GLM_2_.

To investigate our secondary hypothesis that individual differences in pessimism explain ambiguity aversion in Version A of the paradigm and outcome expectancy in Version B, we refit the models above and added models including the factor pessimism to a subset of the data (*n* = 64) due to incomplete cases. This resulted in 1400 observations for the training set (random subset of 75% of the complete data) and 467 observations for the test set. Refitting the intial models to the subset of participants showed the same pattern as above, i.e. all three null models (A_GLM_0_, B_GLM_0_, and B_GLMc_0_) were improved by the factor uncertainty. Adding the factor pessimism to the uncertainty models improved only the model predicting guesses in Version B of the paradigm (B_GLM_1_), but not the two other models (A_GLM_1_ and B_GLMc_1_), indicating that individual levels of pessimism can explain outcome expectancy, but not ambiguity aversion or confidence. Details of the model comparisons are reported in Table 3. The final model explaining guesses in Version B of the paradigm based on the factors uncertainty and pessimism (B_GLM_2_) predicts with an accuracy of 72.81% on the test set and produces an AUC of 0.79 for the ROC. The parameter estimates for the added factor pessimism can be found in Supplementary Table S5. The fixed effects of pessimism in the models A_GLM_2_ and B_GLM_2_ are plotted in Fig. 3a,b, respectively. As expected, the plot shows a negative linear effect of pessimism on probabilities of guessing “win” in Version B of the paradigm. However, in Version A of the paradigm, the effect of pessimism on probabilities of placing a bet is stronger in the high risk (50%) condition than in the ambiguity and does not affect both low risk conditions. Under the premise that individual levels of pessimism affect ambiguity aversion, one would expect the distance between predicted probabilities in high risk and ambiguity to be smaller for low levels of pessimism and larger for high levels of pessimism. This indicates that individual differences in pessimism can influence weighting of probabilities for both high risk and ambiguity, but that they do not explain ambiguity aversion.

**Table 3:** Binomial logistic regression model comparisons for Version A and Version B on sample subset.

Model	Log Likelihood	AIC	BIC	Pseudo-R^2^	Likelihood ratio test
A_GLM_0_	−889.49	1783.0	1793.5	0.23	Model χ^2^(3) = 761.89, *P* < .001 Model χ^2^(1) = 0.24, *P* = .622
A_GLM_1_	−508.55	1027.1	1053.3	0.79	
A_GLM_2_	−508.43	1028.9	1060.3	0.79	
B_GLM_0_	−928.96	1861.9	1872.4	0.07	Model χ^2^(3) = 261.54, *P* < .001 Model χ^2^(1) = 5.85, *P* = .016
B_GLM_1_	−798.19	1606.4	1632.6	0.32	
B_GLM_2_	−795.27	1602.5	1634	0.32	
B_GLMc_0_	−840.60	1685.2	1695.7	0.18	Model χ^2^(3) = 465.76, *P* < .001 Model χ^2^(1) = 0.11, *P* = .744
B_GLMc_1_	−653.92	1317.8	1344.1	0.49	
B_GLMc_2_	−653.87	1319.7	1351.2	0.49

### Analysis of reaction times

The reaction times in both paradigms were analysed using mixed effects linear models accounting for the repeated measures design.

Model specification for both paradigms (A_ and B_):

y = Reaction times (in milliseconds); x_1_ = Uncertainty; ε = Random effects (Participant)

$${{\rm{LM}}}_{{\rm{0}}}:\,y={\theta }_{0}+\varepsilon $$

$${{\rm{LM}}}_{{\rm{1}}}:\,y={\theta }_{0}+{\theta }_{1}{x}_{1}+\varepsilon $$

For Version A of the paradigm, the linear model including uncertainty as a fixed effect A_LM_0_ significantly improved the null model (χ^2^(3) = 103.21, *P* < .001). The parameter estimates are reported together with their confidence intervals in Supplementary Table S5. Post-hoc pairwise comparisons (see Supplementary Table S6) using least square means revealed that there was a strong trend in the difference between high risk and ambiguity. The difference between 25% risk and 75% risk was, unsurprisingly, not significant. All other contrasts were highly significant. Also for Version B, the linear model with uncertainty as a fixed effect (B_LM_1_) significantly added to the null model (χ^2^(3) = 47.63, *P* < .001). The parameter estimates and confidence intervals are reported in Supplementary Table S7. The post-hoc contrasts showed that only the differences between low risk (25%) and each of the other conditions was significant. See Supplementary Table S8 for detailed results of the post-hoc contrasts. Supplementary Fig. S1 shows the distribution of reaction times for both paradigms together with the significance levels of the different contrasts.

### Participant feedback

Immediately after completing both paradigms, the first set of participants (*n* = 38) were asked to give feedback on the task. They rated “fun” and “similarity to a real game” on 5-point rating scales ranging from “not at all” to “entirely”. Version B was rated higher than version A in both categories (“fun”: Version A M: 2.97 ± 1.15 s.d., Version B M: 3.34 ± 0.99 s.d., *F*(1,74) = 2.23, *P* = .14; “similarity to a real game”: Version A M: 2.45 ± 0.92 s.d., Version B M: 3.32 ± 0.96 s.d., *F*(1,74) = 16.16, *P* = .0001). Comments point mainly towards dissatisfaction and disengagement in Version A of the paradigm due to the missing outcome.

## Discussion

In the present study, we tested the theory that ambiguity aversion is caused by pessimism. Contrary to this hypothesis, the results from our new *Coloured Card Deck Paradigm* suggest that individuals are not overly pessimistic during decision-making under ambiguity. The comparison of the two paradigms rather suggests that under uncertainty, subjects display cautious (or “conservative”^6^) behaviour, possibly arising from learning from previous experience, and not pessimistic behaviour: They prefer known probabilities to ambiguity in Version A, but do not respond overly pessimistically to ambiguity, i.e., do not estimate their chances of winning inferior to 50%, in Version B. This contradicts decision theories^27–29^ that explain ambiguity aversion by pessimism when individuals do not know the probabilities. Investigating the effect of individual differences in pessimism on the responses in both paradigms, we could moreover show that, while pessimism has a negative linear effect on outcome expectancy, it did not explain ambiguity aversion. As a basis for our new paradigm, we first replicated the main findings regarding ambiguity aversion from Hsu, *et al*.^31^. Mainly, we observed a difference between the high risk and the ambiguity condition, which supports the notion that they are treated in different ways. Here, ambiguity was linked to decreased probabilities of placing a bet. In the new paradigm, the ambiguity condition produced slightly more pessimistic guesses than high risk. Nevertheless, all conditions except the one with least chances of winning showed overly optimistic beliefs in participants. Instead of pessimism, the results from the second paradigm therefore point towards skewed priors for different levels of uncertainty: Participants are biased towards guessing “win” in three out of four conditions. This is also reflected by the differences in reaction times. In light of the Dual Systems Theory^3^, we hypothesised reaction times to be positively correlated with the amount of uncertainty. However, while this was true for the first paradigm, the reaction times in the second paradigm were shortest in the condition with 75% chances of winning and longest in the opposite condition (25% chances of winning) – an additional indicator towards skewed prior beliefs of winning. In this perspective, no cognitive dissonance has to be resolved in the 75% risk condition and therefore faster, more intuitive System 1-thinking is employed. In the other conditions, prior optimistic beliefs of winning have to be updated (System 2) and are therefore processed more slowly proportional to the decreasing chances of winning. For both paradigms, the models containing different levels of uncertainty that we fit to the datasets performed above chance when predicting participants’ decisions on a test set. This indicates that the differentiation of ambiguity and risk as two different types of uncertainty suggested by Ellsberg^8^ has both explanatory and predictive relevance for the employed tasks.

The results reported above are subject to certain limitations. The distribution of reaction times shows that there might have been an insufficient number of practice trials, as indicated by longer reaction times at the beginning of each paradigm. This may also have caused noise on responses due to limited understanding of the task. However, due to the randomisation of conditions, this should not have a systematic effect on the statistical analyses other than possibly smaller overall effect sizes. While the findings from the confidence model of the *Coloured Card Deck Paradigm* were surprising, the interpretation is limited, since only two response options (“sure” and “unsure”) were given. If measured on a more sensitive scale, the difference in confidence between ambiguity and high risk could potentially become significant.

Decision-making under uncertainty is an ability that is central to our every-day life. While ambiguity aversion is a common strategy to reduce uncertainty, it seems that, when forced to make a decision, individuals are able to not let their decisions be biased by irrationally pessimistic prior beliefs. In this study, we could show that the new *Coloured Card Deck Paradigm* is a suitable paradigm to investigate optimism and pessimism in decision-making under uncertainty. Our results question the theory that ambiguity aversion can be explained by pessimistic priors. While participants prefer known odds to ambiguity, their outcome expectancies are close to equiprobability under ambiguity when forced to place a bet. This suggests that under known risk, individuals form overly optimistic priors, while ambiguity does not produce pessimistic, but realistic priors. Our findings that individual differences in pessimism can explain outcome expectancies but not ambiguity aversion reinforce the theory of subjective weighting of probabilities as a function of personal attitudes^24^. This refines the observed overly optimistic responses in Version B of the paradigm to illustrate a linear weighting based on levels of pessimism. As adaptive strategies, this observation could reflect different balancing of reward-seeking and “bracing”, i.e. optimistic and pessimistic preparedness, respectively, both of which have been argued to yield evolutionary advantages for a decision-maker^25^. Crucially, our results also point towards a missing link in the current conceptualisation of decision-making under uncertainty. In our study, ambiguity aversion seemed to ensue in the whole sample independent of general pessimism, possibly due to a third factor related to the inability to form a prediction. In this perspective, ambiguity aversion seems to be more motivated by general caution as a result of insufficient information to predict the outcome, while pessimism affects subjective weighting of probabilities independent of these cautious strategies.

## Methods

In the present study, we use the terms risk and ambiguity as different types of uncertainty (knowing and not knowing the odds, respectively) as defined by Ellsberg^8^. In order to model different levels of uncertainty, we adapted the *Card Deck Paradigm*^31^. Additionally, we developed a modified version of the original paradigm to assess optimism/pessimism. All participants completed both paradigms in a randomised order. Subsequent to the task, we collected feedback on both paradigms to evaluate their reliability.

In version A of the paradigm (*Card Deck Paradigm*), we adapted the original task from Hsu, *et al*.^31^ aiming at understanding the difference in responses to ambiguity and risk with minor changes: We added a 50% risk condition, which was not included in the original study^31^ (Supplementary Material). Thus, our design tested if participants react differently to ambiguity than to high risk. Instead of random probabilities throughout the task, we employed only three categories of uncertainty: low risk (25% or 75%), high risk (50%), and ambiguity (?-?). In the analysis, we subdivided the low risk condition into 25% (of blue cards) and 75% (of blue cards) to identify a possible confounding effect of colour. The different uncertainty conditions are illustrated in Fig. 1b. The same trials were repeated a total amount of 10 times within each condition. The monetary gain in the case of a correct bet and opt-out was fixed at 10 € and 3 €, respectively. In this way, we could avoid that the amount of possible gain confounds the behaviour, as described by utility theory^32^. No additional changes were made to the paradigm.

Each trial lasted 5500 ms (time-out). Trials were separated by a 500 ms break screen. This was to ensure that the onset of each new trial was visually salient, since otherwise only the numbers inside the boxes changed. In this way, the duration of a single trial resulted in 6000 ms for each paradigm. An example trial of the Card Deck Paradigm is illustrated in Fig. 1a. The resulting 30 trials were divided into two blocks of 15 trials each. Between blocks, participants were shown a fixation cross of 6000 ms duration. The three levels of uncertainty were randomised throughout the experiment. Response options were mirrored (right-left) for half of the sample to minimise motor effects.

Immediately prior to the experiment, participants were presented with the instructions (incl. screenshots from the task, no time-out) and completed seven practice trials. During this time, they were able to ask questions related to the correct understanding of the task.

For version B of the paradigm, we employed the same card decks and uncertainty categories (see Fig. 1e). Participants were assigned a colour. We randomised which of the two colours (the top or the bottom colour) is assigned. Instead of a bet/opt-out question, we asked participants whether, given the indicated probabilities, they believed they were going to win or lose, as well as whether they felt sure or unsure about their prediction. Importantly, in this version of the paradigm, the outcome of each trial was shown to the participants to increase task engagement. In order to avoid counting, we then changed the colours within the card deck among 10 different pairs. Details on the employed colours can be found in Supplementary Table S9. The trial duration was similar to version A (6000 ms for the decision-making) with an additional 2000 ms to see the outcome. Figure 1d illustrates an example trial of the Coloured Card Deck Paradigm.

The design of uncertainty conditions and trial blocks was identical to version A of the paradigm. Additionally, the orders of colour pairs as well as outcome were randomised. The frequencies of each outcome were in line with the indicated probabilities, and set to 50% for the ambiguity condition. Like in Version A, response options were mirrored (right-left) for half of the sample. The design of instructions and practice trials was similar to Version A of the paradigm.

The data was collected with two groups of participants according to the two different data collection devices: Participants belonging to sample 1 completed the paradigm at a computer in the laboratory studio and responses as well as reaction times were collected from the keyboard. Participants in sample 2 completed the experiment inside an MRI scanner and responses as well as reaction times were collected on an MR-compatible four-finger button press. The neuroimaging data analysis is currently ongoing and will be reported elsewhere. Sample size was determined through power calculation to have sufficient power (0.8) to detect differences in each of the two samples based on the effect sizes reported in the original study^31^. We recruited 78 participants overall, among which 34 were female (Sample 1: 40 participants; 19 female; Sample 2: 38 participants; 15 female). In the second sample, two participants only completed one of the two paradigms (Version A or Version B) due to late arrival on the scheduled test day. All other participants completed both paradigms, resulting in *n* = 77 for each of the paradigms. Participants were between 18 and 51 years old with a mean age of 27.52 ± 5.91 s.d. (Sample 1: M: 27.65 ± 5.79 s.d.; Sample 2: M: 27.40 ± 6.10 s.d.). We recruited participants from a variety of socio-economic backgrounds. We screened for the exclusion criteria colour blindness (because the paradigm relies on coloured cards), drug or alcohol abuse via the Addiction Severity Index^33^, and pathological gambling disorder via the South Oaks Gambling Screen^34^.

The study was approved by the internal review board of the Faculty of Medicine at Technische Universität Dresden (IRB00001473/IORG0001076). Each participant was informed about the data and insurance policy and voluntary nature of the study, according to the Declaration of Helsinki^35^. They were asked to confirm by signature their consent to the conditions of their participation in the study. The paradigms were presented with OpenSesame^36^. All participants completed both paradigms in a random order. Between paradigms, participants were shown a break screen (fixation cross) of one minute. In order to generate enough data points to reliably train and test the models, the two samples were collapsed for the analysis. Immediately before the experiment, we assessed the Positive and Negative Affect Scale^37^. Subsequent to the experiment, we collected the Short Scale for the Assessment of Risk-Taking^38^, the Scale Impulsive Behaviour^39^, and the Scale Optimism/Pessimism^40^ to allow linking personality-related predispositions regarding the preference for and processing of uncertainty to our findings. 64 participants completed this part of the data collection.

We recorded button presses and reaction times. The statistical analysis of the behavioural data was carried out in R^41^. In order to allow comparing the findings from the two paradigms, we treated the low risk condition of the first paradigm as two conditions based on the amount of red cards (75% and 25%, respectively). Since we only examined responses in terms of placing a bet, these two conditions should produce the same results. After removing missing values, the datasets used for the models consisted of 2225 observations for Version A and 2322 observations for Version B. To build and evaluate the models, the original datasets were randomly split into a training set (75% of the data) and a test set (25% of the data). Using mixed effects binomial logistic regression modelling, we investigated whether the decision-making in the paradigm can be explained by the different types of uncertainty (ambiguity, high risk, and low risk). The models were fitted using the numeric BOBYQA-optimiser algorithm implemented in the R-package lme4^42^. The specified models were then compared with likelihood ratio tests. We used the parameters obtained from the winning model to predict responses on the test set to validate the performance of the model. Based on significant main effects, we finally computed Bonferroni-corrected post-hoc pairwise comparisons between the conditions using least square means. This procedure was applied to both versions of the paradigm. The raw responses are shown in Fig. 1c for Version A of the paradigm and Fig. 1f for Version B of the paradigm. Reaction times were analysed using linear mixed effects models with Bonferroni-corrected post-hoc least square means comparisons. The raw reaction times are plotted together with their distribution in Supplementary Fig. S1.

## Supplementary information

Supplementary material

## Data Availability

The datasets generated and analysed during the current study are available from the corresponding author on reasonable request.
