# Supplementary material for: Pessimistic outcome expectancy does not explain ambiguity aversion in decision-making under uncertainty
Source: Sci Rep. 2019 Aug 21;9:12177. doi: 10.1038/s41598-019-48707-y (PMC6704180; doi:10.1038/s41598-019-48707-y)
Supplement: Supplementary file 1 — Supplementary material [file 41598_2019_48707_MOESM1_ESM.pdf]

Supplementary material

Pessimistic outcome expectancy does not explain ambiguity aversion in decision-making under uncertainty

Ahrends C*, Bravo F, Kringelbach ML, Vuust P, Rohrmeier MA

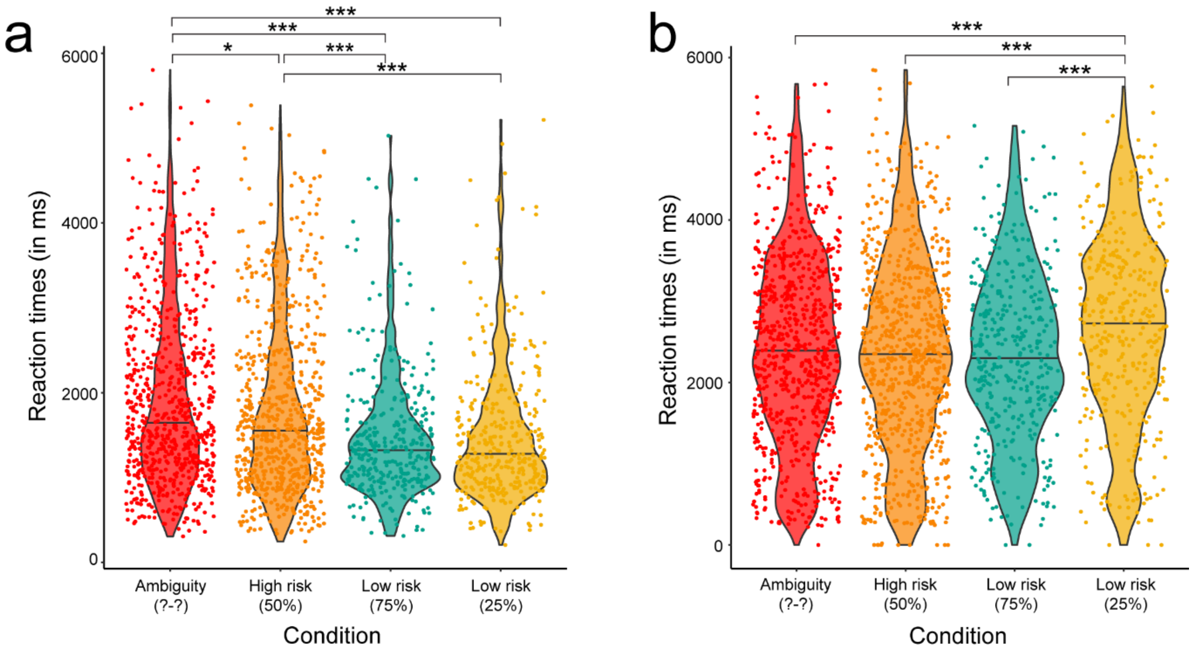

Supplementary Figure S1 Violin plots of reaction times (y-axis, in milliseconds) by uncertainty condition (x-axis) for Version A (a) and Version B (b) of the paradigm. *The violin bodies illustrate the distribution of reaction times over the possible trial duration (6000 milliseconds). The horizontal lines inside the bodies are the medians of the data. The scatterplots show the actual observed values. The significance levels indicated by asterisks are obtained through pairwise comparisons of least square means with Bonferroni correction.* ***(a)*** *shows that the length of reaction times follows the level of uncertainty with high uncertainty (ambiguity condition) producing longer reaction times than low uncertainty (low risk 25% and 75%). All comparisons except the within low risk-subdivision are significant.* ***(b)*** *illustrates that the low risk condition with 25% chances of winning produces significantly longer reaction times than all other conditions. The comparisons between ambiguity, high risk, and low risk (75%) are not significant. The reaction times are overall longer for Version B* ***(b)*** *than for Version A* ***(a)****.*

Supplementary Table S2 Obtained weights and odds ratios for fixed effect uncertainty from Version A model A_GLM$_1$

	**Parameter estimate**	**Standard Error**	**95% Confidence Interval for Odds Ratio**		
			Lower	*Odds Ratio*	*Upper*
(Intercept)	0.34	0.28	0.81	1.40	2.43
Ambiguity	-2.27***	0.18	0.07	0.10	0.15
25% risk	4.57***	0.45	42.54	96.76	251.75
75% risk	3.77***	0.37	21.75	43.23	94.22

Supplementary Table S3 Obtained weights and odds ratios for fixed effect uncertainty in Version B from model B_GLM_1

	**Parameter estimate**	**Standard Error**	**95% Confidence Interval for Odds Ratio**		
			Lower	*Odds Ratio*	*Upper*
(Intercept)	0.70	0.12	1.58	2.01	2.57
Ambiguity	-0.34**	0.13	0.55	0.71	0.92
25% risk	-2.10***	0.18	0.09	0.12	0.17
75% risk	1.93***	0.24	4.38	6.88	11.26

Supplementary Table S4 Obtained weights and odds ratios for fixed effect uncertainty from Version B model B_GLMc_1

	**Parameter estimate**	**Standard Error**	**95% Confidence Interval for Odds Ratio**		
			Lower	*Odds Ratio*	*Upper*
(Intercept)	-1.68***	0.17	0.13	0.19	0.26
Ambiguity	-0.29	0.17	0.54	0.75	1.04
25% risk	2.33***	0.18	7.23	10.31	14.87
75% risk	2.74***	0.19	10.62	15.41	22.70

Supplementary Table S5 Obtained weights and odds ratios for fixed effect pessimism from Version B model B_GLM_2

	**Parameter estimate**	**Standard Error**	**95% Confidence Interval for Odds Ratio**		
			Lower	*Odds Ratio*	*Upper*
Pessimism	-0.19*	0.08	0.70	0.82	0.96

Supplementary Table S6 Obtained parameter estimates for fixed effect uncertainty in reaction time model Version A

	**95% Confidence Interval for Parameter Estimate**			**Standard Error**	**t-value**
	Lower	*Estimate*	*Upper*		
(Intercept)	1664.45	1795.23***	1926.02	66.73	26.90
Ambiguity	27.87	110.49**	193.10	42.15	2.62
25% risk	-408.34	-307.32***	-206.29	51.54	-5.96
75% risk	-408.69	-307.29***	-205.88	51.74	-5.94

Supplementary Table S7 Post-hoc comparisons using least-square means for reaction time model Version A (p-values and confidence intervals adjusted using Bonferroni correction)

**Contrast**	**95% Confidence Interval for Estimate**			**Standard Error**	**t-ratio**	**p-value**
	Lower	*Estimate*	*Upper*			
High risk (50%) – Ambiguity (?-?)	-221.80	-110.39	0.83	42.15	-2.62	.05
High risk (50%) – Low risk (25%)	171.20	307.32	443.43	51.54	5.96	***<.0001
High risk (50%) – Low risk (75%)	170.66	307.29	443.92	51.74	5.94	***<.0001
Ambiguity (?-?) – Low risk (25%)	281.87	417.80	553.74	51.48	8.12	***<.0001
Ambiguity (?-?) – Low risk (75%)	281.30	417.77	554.24	51.58	8.08	***<.0001
Low risk (25%) – Low risk (75%)	-157.37	-0.03	157.31	59.58	0.00	1.00

Supplementary Table S8 Obtained parameter estimates for fixed effect uncertainty in reaction time model Version B

	**95% Confidence Interval for Parameter Estimate**			**Standard Error**	**t-value**
	Lower	*Estimate*	*Upper*		
(Intercept)	2165.75	2356.41***	2547.07	96.80	24.34
Ambiguity	-20.82	60.24	141.31	41.37	1.46
25% risk	224.35	324.04***	423.73	50.88	324.04
75% risk	-122.02	-22.51	77.00	50.78	-22.51

Supplementary Table S9 Post-hoc comparisons using least-square means for reaction time model Version B (p-values and confidence intervals adjusted using Bonferroni correction)

**Contrast**	**95% Confidence Interval for estimate**			**Standard Error**	**t-ratio**	**p-value**
	Lower	*Estimate*	*Upper*			
High risk (50%) – Ambiguity (?-?)	-169.49	-60.24	49.01	41.37	-1.46	.87
High risk (50%) – Low risk (25%)	-458.39	-324.04	-189.69	50.88	-6.37	***<.0001
High risk (50%) – Low risk (75%)	-111.59	22.51	156.61	50.78	0.44	1.00
Ambiguity (?-?) – Low risk (25%)	-398.03	-263.80	-129.57	50.83	-5.19	***<.0001
Ambiguity (?-?) – Low risk (75%)	-51.24	82.75	216.74	50.74	1.63	.62
Low risk (25%) – Low risk (75%)	191.41	346.55	1501.69	58.75	5.90	***<.0001

Supplementary Table S10 Employed colours in Version B (Coloured Card Deck Paradigm)

**Colour 1**		**Colour 2**	
Name	*HEX-code*	*Name*	*HEX-code*
Satin sheen gold	#cd962c	Lapis lazuli	#257195
Dark blue	#000093	KU Crimson	#e80308
Shamrock green	#059c57	Golden brown	#976728
Orange-red	#fd4403	Plum (traditional)	#942495
Red (Munsell)	#e7003d	Denim	#2473b9
Burgundy	#850031	Deep saffron	#fea52e
Cyan (Process)	#04aeee	Vivid cerise	#e20286
Falu red	#89141c	Mantis	#6fc44d
Titanium yellow	#f3e700	Bulgarian rose	#560000
Bistre	#3d2316	Apple green	#84d600
